# Initial Biomechanical Properties of Transtibial Meniscal Root Repair are Improved By Using a Knotless Anchor as a Post-Insertion Tensioning Device

**DOI:** 10.1038/s41598-020-58656-6

**Published:** 2020-02-04

**Authors:** Maria Prado-Novoa, Ana Perez-Blanca, Alejandro Espejo-Reina, Maria Jose Espejo-Reina, Alejandro Espejo-Baena

**Affiliations:** 1 Laboratory of Clinical Biomechanics, Málaga, Spain; 2 Department Mechanical Engineering, Universidad de Málaga, Andalucía Tech, Málaga, Spain; 3 Hospital Vithas Parque San Antonio, Málaga, Spain

**Keywords:** Preclinical research, Biomedical engineering, Mechanical engineering

## Abstract

The importance of meniscal root integrity to preserve contact load distribution and stability at the knee joint is recognised. Transosseous suture technique is commonly used to repair meniscal root tears. However, clinical results are not completely satisfactory. Specifically, concern exists about the development of substantial displacements at the repaired root. This study aims to assess if the use of a post-insertion tensioning knotless-anchor at the distal exit of the tibial tunnel improves time-zero biomechanical properties of the transtibial repair compared to knotting sutures over a cortical button. Twenty porcine tibia with detached posterior medial meniscal roots were randomized into two groups depending on the method to fix the sutures after root repair: knotless-anchor (KA) or suture-button (SB). Specimens underwent cyclic and load-to-failure testing. Group KA showed significantly smaller residual root displacements after low-level repetitive loads. At the load-to-failure test, Group KA exhibited significantly lower displacements at representative subcritical loads and higher resistance to development of clinically relevant displacements. The authors conclude that use of a knotless suture anchor attached at the distal outlet of the bone tunnel may be an effective solution to reduce root displacements in transtibial meniscal root repairs, a matter reported to alter biomechanics of joint contact.

## Introduction

Root repairs are common as root tears have been increasingly recognized. Biomechanical studies confirmed the importance of meniscal root integrity to preserve joint contact load distribution^1–4^ and stability^5^. Meniscal root repair surgery aims to re-establish these meniscal functions^6,7^ and to minimize progression of osteoarthritis^8^.

Transosseous repair is the preferred choice for many surgeons, because it is reproducible, facilitates access to the anatomical insertion area and may enhance meniscal healing by allowing marrow content into the articular space. However, clinical results of this technique are not completely satisfactory. Specifically, substantial displacements at the repaired root have been reported in biomechanical studies under cyclic loads simulating postoperative rehabilitation^9,10^, a situation expected to alter biomechanics of joint contact as observed in a non-anatomical root repair^11,12^. Repair fixation needs to be optimized and reducing root displacements should be one of the main goals.

A recent work monitored the meniscus-suture interface and found no macroscopic damage at the meniscus-suture boundary up to a load close to ultimate failure^13^. This implies that sources other than suture cutout should contribute to generating permanent displacements, such as knot squeezing or slipping, since a certain degree of knot slippage under load has been reported^14,15^. In transtibial repair techniques, sutures are frequently fixed by tying them over a cortical button or a post at the distal end of the bone tunnel exit. Some works have recently described the use of knotless anchors to secure the sutures at the distal exit of the tunnel^16,17^. However, available suture anchors are routinely used for attachment of soft tissue to bone in shoulder procedures; the adequacy of its use for transtibial meniscal root refixation should be assessed.

The purpose of this study was to compare, in an *in vitro* porcine model, the initial biomechanical properties of transtibial root repairs using two different methods to fix the sutures: knotting them over a suture button (SB) or with a knotless anchor (KA) that allows post-insertion tensioning attached to the anterior tibial cortex. The study hypothesis was that the KA method provides lower displacements and higher resistance than the SB method.

## Materials and Methods

Twenty tibiae and its medial menisci from fresh frozen porcine knees of 5 month old animals (approximately 100 Kg) donated by a local authorized butcher were included in this study. No ethical approval was required for material generated as waste from food production.

### Specimen preparation

**Figure 1:**
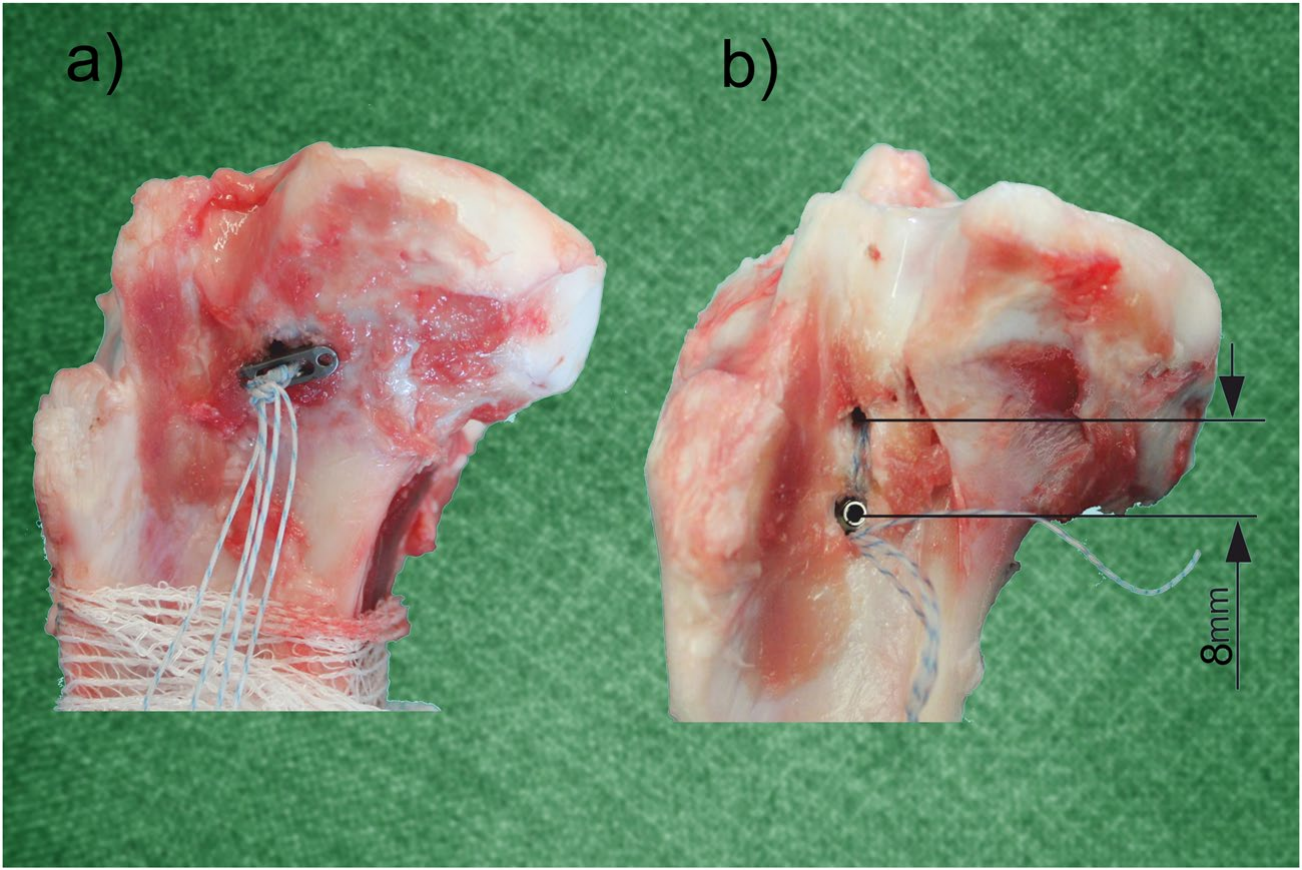
Study groups depending on the distal suture fixation method (lateral view of left porcine knees): (**a**) Group SB - Sutures are knotted over a button; (**b**) Group KA: sutures are inserted in a knotless fixation device that is impacted at 8 mm distal to the tunnel exit.

The specimens were prepared as in previous works of the group^13^, the knees were individually packed in sealed plastic bags and frozen at −20 °C. The day before the test of a specimen, a knee was left at room temperature. After thawing, it was cleaned of muscles and soft tissue and the tibia was extracted with the medial meniscus attached prepared for the test. If the tibia/meniscus complex met the inclusion criteria of absence of any macroscopic sign of degeneration or injury, it was randomly assigned to a study group depending on the suture fixation method employed at the tunnel exit: Group SB, sutures knotted over a button (Fig. 1A); Group KA, fixation achieved by means of a knotless anchor system (Fig. 1B).

**Figure 2:**
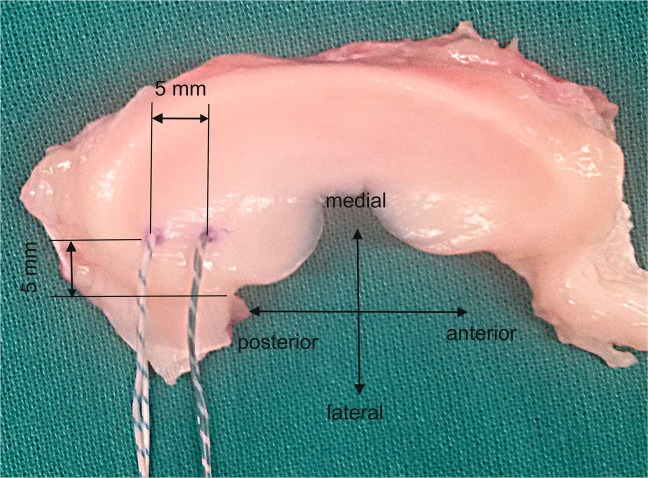
Superior view of a right porcine meniscus. Location of suture entry points at 5 mm from the lateral edge of the medial meniscus and separated at 5 mm.

The same expert surgeon conducted all the surgical procedures. Firstly, the posterior root was transected. A 5 mm tunnel was drilled from the anterolateral tibial epiphysis to the posterior root insertion site^4^. Two ultra-resistant N. 2 sutures (Force Fiber™, Stryker, Endoscopy, San José CA) were applied in a two-simple-stitch fashion, an easy suturing method previously used by our group^13,16^ with proven potential to resist displacement, which is widely used^18^. A simple meniscal suture device previously used for transtibial pullout repairs^16,19^ was loaded with one suture and passed through the tunnel in an outside-in manner to pierce the meniscus. The suture was held and the device removed, leaving a thread loop at the femoral side. One tail of the thread was drawn out of the tunnel while holding the other tail at the anterior exit to form a simple stitch. The process was repeated for a second thread. The suture entry points in the posterior horn were at approximately 5 mm from its lateral edge and separated at 5 mm (Fig. 2). Finally, the meniscal root was brought into or just over the articular end of the tibial tunnel by pulling out the sutures^4^.

In group KA, the anterior cortex of the tibia was perforated with an awl at 8 mm distal to the tunnel (Fig. 1B). Following manufacturer’s instructions, each pair of suture tails was passed through a different pull-tab of a 4.5 mm ReelX® STT knotless anchor (Reelx-STT®, Stryker, Endoscopy, San José, CA), which was then inserted in the hole created. Sutures were tensioned by means of the incremental tensioning mechanism incorporated in the anchor, until the root felt firmly attached in place when pulled with a grasper. In group SB, manual tension was applied until a similar tension level while tying them in matched pairs over a surgical button (Versitomik G-Lok No loop®, Stryker, Endoscopy, San José CA) using a surgeon’s knot followed by six half-hitches on alternating posts (Fig. 1A). The surgeon specifically sought to achieve a similar level of tension for all the specimens in both groups.

### Biomechanical testing

**Figure 3:**
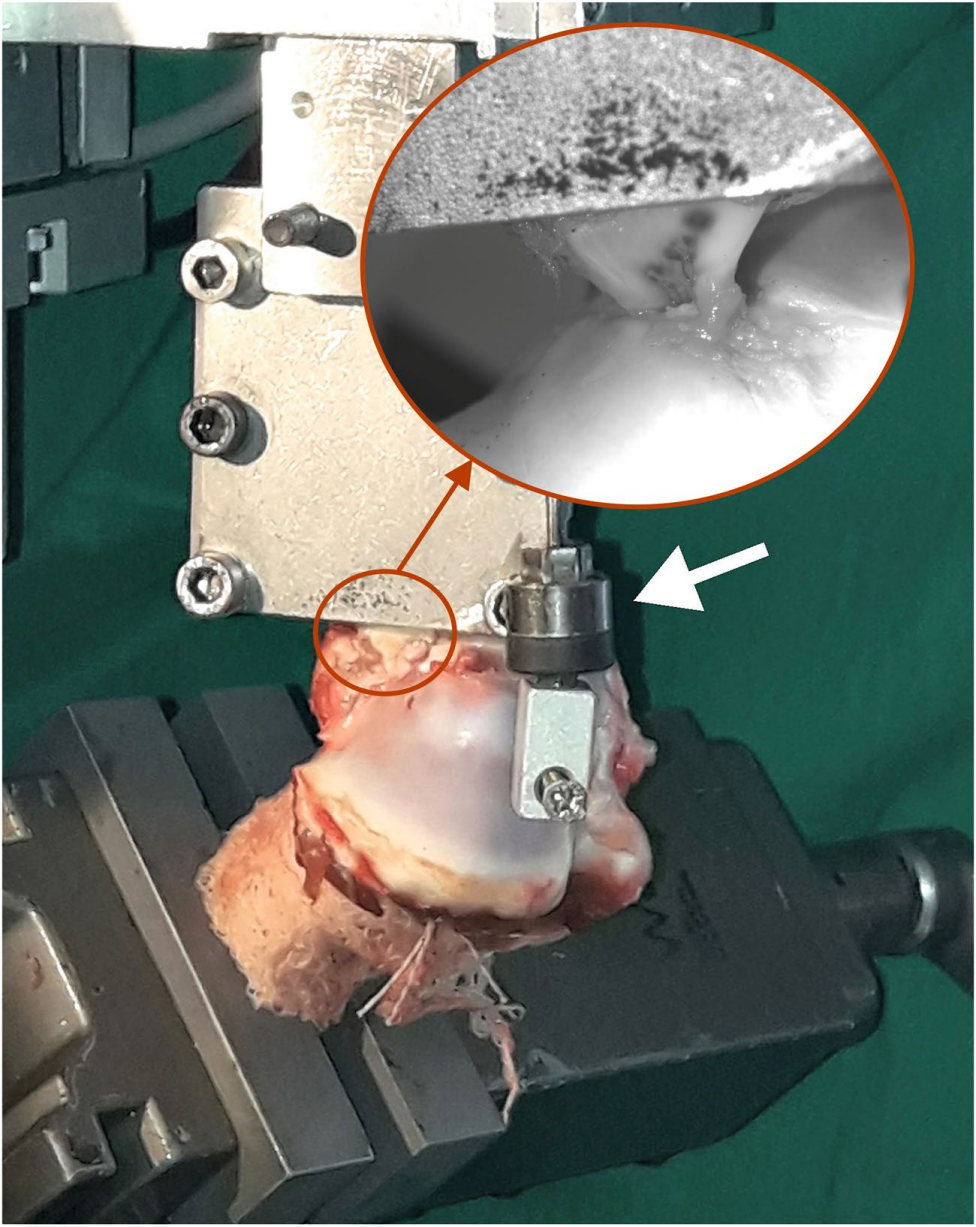
Single-axis testing machine with the specimen mounted. The white arrowhead points to the inductive sensor connected between the head of the machine and a point on the proximal tibia close to the exit of the tunnel to exclude tibial deformation from the recorded displacements. Magnification of the grasp of the meniscus by the clamp of the testing machine is shown (red circumferences).

Tests were performed with a custom made single-axis machine (Fig. 3) previously used in biomechanical studies^4^. The tibiae were cut axially at 10 cm below the joint line and fixed to the base of the machine setting the bone tunnel parallel to the pull direction, in order to focus tensile force on the fixation. The meniscus was clamped to the head of the testing machine at approximately 5 mm from the insertion points of the sutures, with sutures visually aligned with the longitudinal fibres of its posterior root and both parallel to the loading direction. To check for slippage, the tissue was marked with a straight line just below the edge of the clamp.

After preconditioning the specimen with 10 cycles of a load[1–10]N at 0.1 Hz^9,18^, it was loaded for 1000 cycles[10–30]N at 0.5 Hz. This load range approximates tensile forces at the meniscal root under 500 N of joint compression in neutral rotation in a [0°–90°] range of flexion^11^. A finite element model^20^ computed this level of tibio-femoral compression for open-kinetic-chain flexion-extension exercises bearing the weight of the leg and the foot. 1000 cycles was selected as representative of the first post-operative week, before the biological process of healing starts to significantly alter the mechanics of the fixation, planning 150 exercises per day at a normal rate of 1 Hz. If a specimen survived the cyclic test, it was again loaded at 5 N for 10 s and finally loaded-to-failure at 0.5 mm/s. The selected test setup allow for comparison with other published biomechanical studies on meniscal root repair techniques^9,10,21^.

Force was measured by a 1000 N force transducer of accuracy class 0.1 (U2B, HBM, Darmstadt, Germany) and the displacement by an inductive sensor (Micro-Epsilon, Ortenburg, Germany) with a resolution of 0.03 mm. To exclude bone deformation from the measurements, one side of the sensor was attached to the machine head and the other to the proximal region of the tibia (Fig. 3). All signals were sampled at 50 Hz.

For data processing, after applying the Chauvenet’s criterion to account for outliers, a 10-point symmetric moving average filter was applied to the displacement measurements. Root displacements in a cycle were computed at minimum and maximum load (10 N, 30 N) as the difference between sensor measurements immediately after that cycle and in the first cycle. Specifically, values in cycles 100, 500 and 1000 were analysed.

Stiffness (as defined by Feucht *et al*.^22^) was determined. Since a constant stiffness value was considered too limited due to the non-linear load-displacement response, displacements at representative subcritical loads of 50, 75 and 90 N were calculated to characterize the deformation under load of the complex. 50 N was selected as representative of peek tension on the meniscal root of a normal subject during open-kinetic-chain exercises against a resistant force of 30 N acting at the ankle^20^ assuming a linear relationship between tension on the root and tibio-femoral compression^11^, 75 N is representative of the peak load when sitting down and 90 N during gait^23^. Ultimate failure load (UFL) and its associated displacement were also computed. Since displacements at UFL resulted quite large, the loads which produce elongations of 3 mm and of 5 mm were calculated as an alternative measure of the resistance. Previous studies reported these displacements as the threshold above which meniscal function is altered in porcine (3 mm)^24^ and in human models (5 mm)^12^. Finally, the mode of failure was determined.

### Statistical analysis

Using G*Power 3.1.9.2^25^, a minimum sample size n = 10 was estimated as necessary to detect a 0.6 mm difference in root displacements with a two-tailed independent measure t-test, at α = 0.05 and (1-β) = 0.85, assuming equal standard deviations(sd) = 0.4 mm^10^). This difference represents 20% of the 3 mm displacement^24^ and was considered noticeable improvement for the repair technique.

For each group, continuous variables were described using means and sd. After Kolmorov-Smirnov tests confirmed normality of all variables (p > 0.05), two-tailed independent measure t-tests were conducted to evaluate differences between groups. p ≤ 0.05 were regarded as significant.

## Results

Bone tunnel length resulted similar in both groups with a mean value of 39.53 mm (sd 1.92) for group SB and of 38.80 mm (sd 2.23) for group KA, which allowed disregarding the influence of suture length.

One specimen of group KA slipped into the clamp and was not included in the calculation. All the other specimens survived the cyclic test.

Root displacements resulted significantly smaller for group KA at minimum and maximum load after 100, 500 and 1000 cycles (Table 1), becoming double for the suture button at both load levels at the end of the test

**Table 1:** Root displacement (mm) during cyclic tests. SB: Suture Button; KA: Knotless Suture Anchor.

		Group SB		Group KA		p-value
		mean	sd	mean	sd	
10 N	100 cycles	0.64	0.20	0.31	0.15	0.001*
	500 cycles	1.20	0.27	0.53	0.25	<0.001*
	1000 cycles	1.46	0.29	0.66	0.31	<0.001*
30 N	100 cycles	1.52	0.44	0.73	0.28	<0.001*
	500 cycles	1.97	0.46	0.94	0.35	<0.001*
	1000 cycles	2.22	0.49	1.10	0.40	<0.001*

*Significant difference. Differences are considered significant at p > = 0.05.

**Figure 4:**
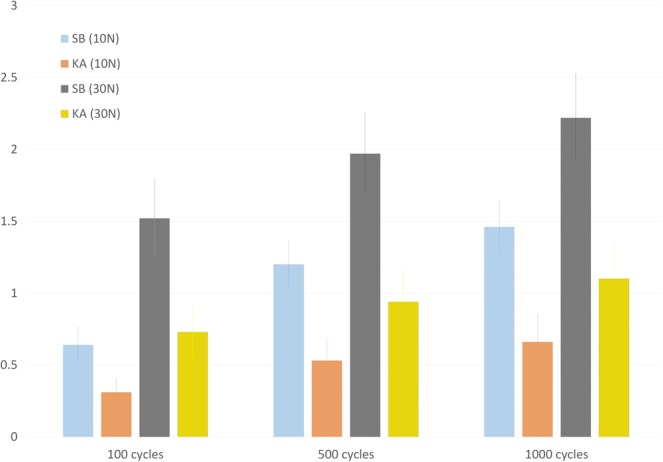
Residual displacement (mm) accumulated during the cyclic tests. Vertical bars represent 95% confidence intervals. SB: Suture Button; KA: Knotless Suture Anchor.

In both groups, over 40% of the cyclic displacement at minimum load and over 60% at maximum load was generated in the first 100 cycles. As the test proceeded, values rose continuously showing an attenuation of the increment per cycle (Fig. 4). The attenuation was more pronounced for group KA, which progressively increased the differences between groups.

Mean stiffness resulted 49.0% higher for group KA. In accordance, displacements at 50 N, 75 N and 90 N were significantly smaller for this group (Table 2).

**Table 2:** Results of the load-to-failure test.

	Group SB		Group KA		p-value
	mean	sd	mean	sd	
Stiffness (N/mm)	26.38	7.25	40.94	9.46	0.003*
Displacement at 50 N (mm)	1.86	0.49	1.04	0.26	<0.001*
Displacement at 75 N (mm)	3.08	0.70	1.81	0.44	<0.001*
Displacement at 90 N (mm)^Ϯ^	4.24	1.17	2.54	0.77	0.003*
Load at 3 mm^ϮϮ^ (N)	74.30	13.75	99.17	12.87	0.001*
Load at 5 mm^ϮϮϮ^ (N)	91.19	19.39	117.63	21.19	0.023*
UFL(N)	108.92	29.38	138.64	30.01	0.053
Displacement at UFL (mm)	5.50	1.64	8.04	4.50	0.159

SB: Suture Button; KA: Knotless Suture Anchor; UFL: Ultimate Failure Load.

*Significant difference. Differences are considered significant at p >  = 0.05.

^Ϯ^1 specimen in group SB did not reach 90 N and was not included in the calculations.

^ϮϮ^In 1 specimen of group SB the displacement at UFL was below 3 mm and it was not included in the calculations.

^ϮϮϮ^In 2 specimens of group SB and in 2 specimens of group KA, the displacement at UFL was below 5 mm and they were not included in the calculations.

All specimens failed by suture pullout through the meniscus. Displacement of the fixation device for group KA or penetration of the button into the bone for group SB were not observed. The mean UFL was 32.5% higher for group KA although it occurred at a mean displacement clearly above the 3 mm threshold for both groups. Group KA was significantly more resistant to elongation, with values of about 30% higher for the mean forces required for elongations of 3 mm and 5 mm (Table 2).

Complete results for all the specimens are made publicly available in the supplementary material.

## Discussion

The most important finding of the present study was that the use of a post-insertion tensioning knotless anchor to fix the sutures outperformed the time zero biomechanical properties of a transtibial repair after a complete tear of the posterior meniscal root compared to tying the sutures over a cortical button. Specifically, the use of the knotless anchor showed a reduction in root displacements developed after low level cyclic loading and an increase in stiffness, which implies a reduction of the displacements under subcritical loads. It also exhibited a higher resistance to developing clinically significant root displacements under load-to-failure testing.

The use of the knotless anchor outperformed the suture button since the first cycles of the test, where cyclic displacement showed the greatest increase. As the test proceeded, the percentage difference between both groups was more pronounced. After 1000 cycles, simulating open-kinetic-chain flexion-extension exercises, the displacement at peak load reached 74% of the 3 mm threshold^24^ for group SB and only 22% for group KA. This displacement approaches the threshold for group SB. We consider that the important reduction found with the use of the KA is worth attention from a clinical perspective.

In the load-to-failure test, the repair resulted stiffer for group KA. However, since the load-displacement curve showed a clear non-linearity, we also compared displacements at representative loading levels, resulting significantly lower displacements for group KA at all studied levels. The loads were selected as representative of aggressive rehabilitation protocols, which program flexion-extension exercises against a resistant force^20^ or even gait^23^. 5 mm has been reported as a displacement threshold that alters meniscal functionality due to a non-anatomic root repair in a human model^12^. Group KA showed displacements reasonably below this threshold even if the cyclic displacement at minimum load, which can be considered as non-recoverable, is added. Although further investigation is necessary to extrapolate the results to the clinical setting, these are promising outcomes to allow early aggressive rehabilitations.

UFL is representative of the resistance of the repair to unexpected overloads. Since it occurred at very large displacements for all the specimens, the authors considered that UFL alone was a poor criterion to represent clinical failure. Therefore, the loads required to generate elongations of 3 mm and 5 mm were computed, values reported to significantly alter knee contact in non-anatomic repairs^12,24^. At both displacement levels, forces were up to 30% higher for group KA. This finding, along with the displacements at the studied load levels, could be of special interest in order to prevent damage of the repaired meniscus caused by unexpected overloads.

Menisci often fail in loading/rotation movements that impose on the posterior meniscal horn high traction loads acting in the direction of root fibers. A single axis testing without soft tissues does not reproduce the natural knee movements but the experimental setup was designed to apply on the repair system a traction that represents a worst-case scenario during the immediate post-operative period, although it does not seek to establish its behavior after this period. It is possible that both techniques compared may not be ideal under natural circumstances.

To our knowledge, this is the first study that evaluates the biomechanical characteristics of meniscal root repairs with knotless suture anchors. Prior biomechanical studies in porcine models with similar testing protocols have analysed transtibial repair techniques using cortical buttons to fix the sutures^9,10^. Cerminara *et al*.^9^ measured a 3.28 mm mean permanent displacement at peak load after 1000 loading cycles. Important differences in the testing protocol may have contributed to obtaining such high values compared to ours. They used longer sutures, higher length of meniscal tissue, included bone deformation in the displacement measure and their suture material provides lower knot slippage resistance than the sutures in our work^26^.

Another work compared an *in-situ* suture anchor repair and a transtibial technique with sutures tied over a titanium plate in a porcine model^10^ In the transtibial pullout repair, the mean permanent displacement registered after 1000 cycles of a [5-20] N load was 2.2 mm with the transtibial technique. Again, notorious methodological dissimilarities difficult contrast of the result with our study. However, when compared with tying sutures over a cortical button at the bone tunnel outlet, results within each study showed analogous mean displacement reductions in their work by tying shorter sutures fixed at the anatomical insertion site with a suture anchor (40.91%) as we found by fixing longer sutures with a knotless anchor at the anterior bone cortex (46.58%),

Stiffness computed from the load-deformation of group SB was similar to the 23.7 N/mm reported in the study of Feucht *et al*.^10^. with a porcine model. As for the UFL, our value of 109.8 N was in the range found by other authors when using a suture button in porcine specimens (180.1 N^10^ and 96 N^21^), although the influence of differing factors like loading direction and suturing technique preclude direct comparison.

The results of the present work are influenced by intrinsic limitations to *in vitro* studies. It does not reflect long-term outcomes.

To focus the study on the meniscus-suture interaction it was separated of the surrounding soft tissue. For the same reason and aiming to eliminate shear friction, the bone tunnel that guides the suture was set parallel to the pull direction, although the situation does not reproduce a physiological load.

Porcine models are frequently selected for comparative studies of meniscal repair techniques^9,10,21,24^ because they decrease bias related to specimen while are considered to be functionally close to young adult humans. Thus, the authors assume that for human specimens the comparative outcomes of the work would hold, although obviously not the absolute values.

The selected knotless device allows a post insertion control of the tensioning on the suture. Knotless anchors that do not permit post insertion tensioning should be further studied.

Finally, a two-simple-stitch technique was used due to its easiness and to its capability to limit the displacements^18^. Nonetheless, we think that the comparative outcomes may be also valid with other suturing techniques

## Conclusions

In a transtibial repair of the posterior meniscal root, the use of a post-insertion tensioning knotless anchor firmly attached to the distal outlet of the bone tunnel to fix the sutures, compared with tying them over a cortical button, significantly decreases root displacements developed after low-level repetitive loads, reduces displacements under subcritical loads and exhibits a higher resistance to developing clinically significant root displacements. From a clinical perspective, this may be an effective solution to reduce root displacements, a matter that has been reported to alter biomechanics of joint contact

## Supplementary information

Supplementary information
