# Supplementary material for: Initial Biomechanical Properties of Transtibial Meniscal Root Repair are Improved By Using a Knotless Anchor as a Post-Insertion Tensioning Device
Source: Sci Rep. 2020 Feb 4;10:1748. doi: 10.1038/s41598-020-58656-6 (PMC7000710; doi:10.1038/s41598-020-58656-6)
Supplement: Supplementary file 1 — Supplementary information [file 41598_2020_58656_MOESM1_ESM.pdf]

INITIAL BIOMECHANICAL PROPERTIES OF TRANSTIBIAL MENISCAL ROOT REPAIR ARE IMPROVED BY USING A KNOTLESS ANCHOR AS A POST-INSERTION TENSIONING DEVICE

Maria Prado-Novoa, Ana Perez-Blanca, Alejandro Espejo-Reina, Maria Jose Espejo-Reina, Alejandro Espejo-Baena

CYCLIC TEST

ROOT DISPLACEMENT AT CYCLE N

	100cycles		500cic		1000cic	
**specimen**	**group KA**	**group SB**	**group KA**	**group SB**	**group KA**	**group SB**
**1**	0.33	0.54	0.59	0.94	0.82	1.12
**2**		0.56		1.13		1.35
**3**	0.60	0.78	0.97	1.51	1.13	1.83
**4**	0.18	0.30	0.31	0.72	0.39	0.95
**5**	0.35	0.40	0.57	0.94	0.69	1.36
**6**	0.18	0.90	0.29	1.45	0.39	1.70
**7**	0.25	0.89	0.48	1.54	0.55	1.84
**8**	0.16	0.75	0.24	1.42	0.28	1.67
**9**	0.51	0.61	0.91	1.11	1.19	1.36
**10**	0.22	0.70	0.40	1.23	0.48	1.46
**mean**	0.31	0.64	0.53	1.20	0.66	1.46
**SD**	0.15	0.20	0.26	0.28	0.33	0.30
**IC 95%**	[0.214, 0,405]	[0.521, 0.766]	[0.365,0.691]	[1.027, 1.373]	[0.455, 0,861]	[1.281, 1.648]
**p-value**	0.001		0.000		0.016	

Mean root displacement per cycle

cycles	group KA	group SS
from 0 to 100	0.00309	0.00643
from 100 to 500	0.00055	0.00139
from 500 to 1000	0.00026	0.00053

LOAD-TO-FAILURE-TEST

	Displacement at 30N		Displacement at 50N		Displacement at 70N	
**specimen**	**group KA**	**group SB**	**group KA**	**group SB**	**group KA**	**group SB**
**1**	0.66	2.07	1.18	2.76	1.85	3.73
**2**		0.70		1.38		2.18
**3**	0.75	1.13	1.27	1.85	2.08	2.84
**4**	0.32	0.62	0.68	0.99	1.07	1.60
**5**	0.50	1.22	0.89	2.15	1.37	3.63
**6**	0.32	0.85	0.65	1.50	1.04	† 11.328
**7**	0.49	1.18	1.03	1.88	1.68	2.99
**8**	0.87	1.28	1.40	2.26	1.99	3.23
**9**	0.59	1.28	1.15	1.97	1.90	2.67
**10**	0.65	1.13	1.13	1.85	1.79	2.67
**mean**	0.57	1.15	1.04	1.86	1.64	2.84
**SD**	0.19	0.40	0.26	0.49	0.39	0.67
**IC 95%**	[0.459, 0.689]	[0.897, 1.396]	[0.884, 1.201]	[1.553, 2.164]	[1.402, 1.884]	[2.395, 3.276]
**p-value**	0.001		0.000		0.001	

	Ultimate load		Displacement at Ultimate load	
**specimen**	**group KA**	**group SB**	**group KA**	**group SB**
**1**	121.89	121.39	5.95	6.28
**2**		112.83		3.81
**3**	95.38	177.49	3.75	8.40
**4**	193.40	126.14	8.19	5.43
**5**	111.44	98.33	2.89	5.70
**6**	137.92	60.12	5.24	2.48
**7**	130.58	94.49	14.11	6.83
**8**	149.24	109.17	12.88	5.61
**9**	126.81	98.04	4.32	6.01
**10**	181.05	91.23	15.06	4.44
**mean**	138.63	108.92	8.04	5.50
**SD**	31.64	30.37	4.75	1.64
**IC 95%**	[119.026, 158.243]	[90.097, 127.748]	[5.100, 10.987]	[4.483, 6.515]
**p-value**	0.053		0.159	

	Load at 3 mm		Load at 5 mm	
specimen	group KA	group SB	group KA	group SB
1	76.08	56.56	105.52	94.43
2		92.38		† 74,335
3	89.53	73.90	† 83,333	115.92
4	118.22	94.98	151.21	120.40
5	109.34	60.20	† 69,014	87.25
6	114.48	†† 54,074	136.67	† 50,727
7	101.25	70.65	117.09	70.92
8	87.53	66.20	95.48	66.20
9	99.63	77.71	116.07	93.39
10	96.50	75.85	101.37	81.01
mean	99.17	74.27	117.63	91.19
SD	13.58	13.04	19.98	19.40
IC 95%	[90.758, 107.586]	[65.747, 82.792]	[103.788, 131.475]	[77.746, 104.632]
p-value	0.001		0.023	
